# The Book World of Medicine and Science

**Published:** 1899-08-05

**Authors:** 


### The Book World of Medicine and Science.

Pyorrhœa Alveolaris. By John Fitzgerald, L.D.S. (London: The Medical Publishing Company, Limited. Pp. 62.)

This is an interesting little essay written by an expert on a subject which, although one of the greatest importance, is far too often neglected by the medical practitioner. The author is not a whit too severe when he ridicules the doctor who treats his dyspeptic (and septic) patients by the exhibition of mist. gent. alk. instead of by the purification of the gums. Indeed, we think if anything the clinical importance of pyorrhœa alveolaris is hardly sufficiently insisted on. How many cases of asthma, indigestion, and even "consumption" would clear up if the gums and teeth were properly regarded! The author cannot be accused of writing for the dental specialists; in fact, he is careful to point out how much the general practitioner can do to remedy this condition. Perhaps some sentences are a little ludicrous. We read on page 18, "The patient is said to have a gumboil, and there are all the symptoms of acute apical pericementitis." But we heartily commend Mr. Fitzgerald's essay, and willingly forgive the author these little slips into megalomania.

An Introduction to Dermatology. By Norman Walker, M.D. With a Frontispiece, 29 Plates, and 34 Illustrations in the Text. (Bristol: John Wright and Co. 1899. Price 8s. 6d. net.)

Although the author of this book modestly calls it "An Introduction to Dermatology," all the more common diseases, those which the practitioner is sure to meet with, are discussed with considerable fulness, while those rarer forms which he may only occasionally come across are described with quite sufficient detail for all practical purposes. Indeed, although, in view of the vastness of the field which even one department of knowledge such as dermatology includes, it is quite correct to call such a book as this an introduction, its scope is such that, so far as the ordinary practitioner is concerned, it might as well be called a handbook to the subject. After an introductory chapter on "Classification, Diagnosis, and the General Principles of Treatment," we come to the various abnormalities and diseases to the consideration of which the main part of the book is devoted. So far as classification is concerned the author, while admitting that this is perhaps the greatest trial of everyone who has to teach dermatology, throws in his lot with Unna. It would be as unkind as it would be unfair to make a fun of any classification of skin diseases, the subject being one that can be looked at from so many points of view; still it may be pointed out as showing the oddities into which people are led by their efforts at classification that we find "pediculosis corporis" placed under "Anomalies of Circulation," while pediculosis capitis and pediculosis pubis come under "Inflammations," a heading which also covers scabies, sudamina, eczema, ringworm, alopecia areata, syphilis, and leprosy. There is no doubt that the author is right in saying that under the heading "Inflammation" are found the vast majority of skin diseases, but with such an all-embracing heading, covering, as it does, such a "vast majority" of the cases under consideration, one can hardly avoid feeling that we do not get much further in our classification after all. A classification which includes alopecia areata under its "inflammations," but relegates pityriasis versicolor to a class of "saprophytes," and lupus erythematosus—not merely in its results but in its active stages—to a class of "atrophies," is as yet far from satisfactory. We do not, however, say this as in any way an adverse criticism of the book before us, but merely as emphasising the difficulties which stand in the way of reducing to any clearly cut nosology such complex maladies as diseases of the skin must always be. Putting classification, however, on one side, and considering the book from the clinical standpoint, we have nothing but praise to give it as a careful and well written description of diseases of the skin, their causes, their features, and their treatment. On every page there is evidence of careful observation, and the directions in regard to treatment are sound, practical, and evidently the outcome of a large experience. As is always the case with coloured illustrations, the plates vary considerably in excellence, but, take them altogether, they serve very well to illustrate the points raised in the text. To our taste, however, some of the black and white prints, apparently reproductions of photographs, are even more convincing than those in colours, and we would especially draw attention to those of pemphigus, of rodent ulcer, and of nodular leprosy. On the other hand, the coloured drawings of lupus vulgaris, of scabies, and of acne vulgaris are admirable pictures both from the representative and the teaching points of view. We can thoroughly recommend the book as a manual of skin diseases.

Massage and the Original Swedish Movements. By Kurre W. Ostrom. Fourth edition. (London: H. K. Lewis, 1899. Pp. 168. Illustrated. Price 3s. 6d.)

This little book has evidently, from the fact that a fourth edition is called for, met with a fair measure of success. It treats fully, but with brevity, of two matters; massage as is it commonly understood, and the system of "movements" first devised by Ling. It is written from the standpoint of the practical masseur, and teems with practical points, while illustrative diagrams are freely provided. The therapeutic application of these systems is treated practically, and with unusual restraint. The practitioner who studies this manual ought certainly to readily learn the simpler methods of massage; he at any rate will be more favourably inclined to the prescription of a valuable though much abused means of healing and alleviation. We should more cordially recommend this brochure if the remarks on uterine massage were expunged. The method will, we trust, never be employed in this country.

Aids to the Treatment of the Diseases of Children. By John McCaw, M.D. Second Edition. 240 pp. (London: Baillière, Tindall, and Cox. 1899.)

This little book manages to convey within its 240 closely-printed pages no inconsiderable amount of information concerning the diseases of children, both "medical" and "surgical." Its plan is simple; that is to say, the subjects are arranged in alphabetical order, and hence reference is easy. It is not, as the title might lead one to suppose, a book of treatment alone. The diagnosis, prognosis, symptomatology, and morbid anatomy of each disease are treated of; indeed, "treatment" pure and simple is, if anything, too cursorily dealt with. The information given is, on the whole, accurate, if perhaps conveyed in somewhat old-fashioned terms. There is a complete and accurate index. If the use of this book do not lead a student to forsake the text-books, it will no doubt contrive to fill, as a work for ready reference and the refreshing of memory, a useful place.

